# Assessment of the general public’s knowledge of atrial fibrillation through social media: a cross-sectional study

**DOI:** 10.1186/s12912-023-01378-7

**Published:** 2023-06-16

**Authors:** Brigitte FY Woo, Jeroen M Hendriks, Wilson Tam, Toon Wei Lim

**Affiliations:** 1 Alice Lee Centre for Nursing Studies, Yong Loo Lin School of Medicine, Clinical Research Centre, National University of Singapore, Level 3, Block MD11, 10 Medical Drive, 117597 Singapore, Singapore; 2 Caring Futures Institute, College of Nursing and Health Sciences, Flinders University, Sturt Road, Bedford Park South Australia 5042, GPO Box 2100, Adelaide, SA 5001 Australia; 3 Centre for Heart Rhythm Disorders, University of Adelaide and Royal Adelaide Hospital, Port Road, Adelaide, SA 5001 Australia; 4 Alice Lee Centre for Nursing Studies, Yong Loo Lin School of Medicine, Clinical Research Centre, National University of Singapore, Level 2, Block MD11, 10 Medical Drive, 117597 Singapore, Singapore; 5 National University Heart Centre, National University Health System, 1E Kent Ridge Road, Level 13, 119228 Singapore, Singapore

**Keywords:** Atrial fibrillation, Knowledge, Assessment, Survey, Social media, Digital Marketing

## Abstract

**Background:**

Early detection and timely treatment of atrial fibrillation (AF) remains pivotal to preventing AF-related complications. Public involvement in recognising potential AF symptoms and managing AF is vital for early detection and treatment of AF.

**Objective:**

The aim of the study is to assess the general public’s knowledge of AF using an online survey, disseminated via social media.

**Methods:**

A cross-sectional online survey of the general public was conducted between November to December 2021. The survey’s URL was shared on National University Heart Centre, Singapore’s official Facebook page. Digital marketing strategies were employed to recruit members of the public. The 27-item survey assessed public’s knowledge across five domains: basic information about AF, risk factors of AF, detection of AF, prevention of AF, and management of AF.

**Results:**

The survey involved 620 participants. Approximately two-thirds were between the ages 21 to 40 years (64.5%), female (60%) and had at least a degree (64.7%) as their highest level of education. Participants obtained a mean percentage score of 63.3 ± 26.0 for their AF knowledge. One-way ANOVA was done to examine the associations between the participants’ characteristics and their knowledge of AF. There were no statistically significant differences in the AF knowledge scores across the various sociodemographic subgroups.

**Conclusions:**

Members of the public recruited from Facebook and via digital marketing had moderately good knowledge of AF. However, public awareness pertaining to preventing AF has potential for improvement. The utility of social media in reaching the general public was illustrated through this study.

**Supplementary Information:**

The online version contains supplementary material available at 10.1186/s12912-023-01378-7.

## Background

Atrial fibrillation (AF) is the most prevalent sustained arrhythmia. Globally, there is an estimated 46.3 million people living with AF [1]. With population ageing, the prevalence of AF will only continue to rise. For people above the age of 70 years, the prevalence of AF ranges from 4.6 to 8.2%. Particularly in Asia Pacific, the prevalence of AF has risen more than two-fold over the course of 10 years [2, 3]. The prevalence of AF in Taiwan is estimated to be 4.0% in 2040 and 5.8% in South Korea by 2060 [3]. Based on a community health screening project in Singapore, the AF prevalence of people above the age of 80 years is 5.8% [4].

Ischaemic stroke prevention is a cornerstone of AF management [5]. Notably, 70% of AF-related ischaemic stroke can be prevented by oral anticoagulant (OAC) therapy [6], yet most of AF-related ischaemic strokes happen in patients who are not on OAC therapy. High ischaemic stroke prevalence rates of 10–13% have been reported in the Far East and Southeast Asia [7]. Considering that, early detection and timely treatment of AF remains pivotal to preventing AF-related complications [8]. Late detection and subsequently delayed treatment could be attributed by the general public’s poor knowledge of AF [9]. At present, the global public awareness of AF is poor, the overall awareness of AF can be as low as 25% among the members of the public in some countries [10]. Newly diagnosed and chronic AF patients have also been reported to have poor understanding about their AF condition [11, 12].

Studies in other fields of medicine have shown poor knowledge and awareness of disease among members of the public are significant contributors to poor uptake of community screening programmes [13, 14]. Consequently, public education interventions aimed at improving the public’s AF knowledge may empower people to participate in health screening programmes and seek timely treatment for AF [15]. Nonetheless, prior to developing such public education interventions, it may be advantageous to first explore the learning needs of the public by first assessing their awareness and knowledge of AF.

### Aim

The aim of the study is to assess the general public’s knowledge of AF using an online survey, disseminated via social media.

## Methods

### Study design

A cross-sectional online survey of the general public was conducted, between November to December 2021.

### Setting, recruitment, and participants

The online survey was hosted on Qualtrics, a web-based platform for creating, distributing and collecting surveys [16]. A publicly accessible Universal Resource Locator (URL) of the survey was created. In commemoration of the Global AF Awareness Week 2021, the survey’s URL was shared in Facebook posts on National University Heart Centre, Singapore’s (NUHCS’s) official Facebook page (https://www.facebook.com/NUHCS) on 7 November 2021. In an attempt to recruit members of the public and not just followers of NUHCS’s Facebook page, digital marketing strategies were employed. The posts were advertised to both male and female Facebook users, above the age of 25 years and “lookalikes” whose demographics are similar to people who like and follow the NUHCS Facebook page. This meant that the posts were also appearing on the Facebook feeds of targeted users who were not followers of the NUHCS’s official Facebook page. When these users click on the post, they would be led to the NUHCS’s official Facebook page to attempt the survey.

Convenience sampling was undertaken. Members of the public who visit NUHCS’s official Facebook page could choose to click on the survey’s URL and complete the survey at their own time. They could also share the survey’s URL with their friends (snowball sampling).

The survey’s URL was shared and reposted once weekly for the month of November 2021. Access to the online survey was closed on 30 December 2021. To appreciate the participants for completing the survey, they were reimbursed with five Singapore dollars (SGD) (≈ US$3.6). The participants’ response to the survey is anonymous. However, the participants were requested to leave their contact details if they wished to be reimbursed.

### Instrument

A 27-item survey instrument comprised two sections (Supplementary File 1). Section one consisted of 21 items that assessed the general public’s knowledge about AF. It was adapted from the 21-item Atrial Fibrillation Knowledge Assessment Tool (AFKAT) developed by Abubakar et al. [9]. The AFKAT had five domains: basic information about AF, risk factors of AF, detection of AF, prevention of AF, and management of AF. Each item is a True/False-type statement and the participants were given three options, “True”, “False” or “I don’t know”, to select from. Every correct response received a score of ‘1’ while an incorrect or “I don’t know” response obtained a score of ‘0’, with the total ranging from ‘0’ to ‘21’. The AFKAT is reported to be a valid and reliable instrument, with good internal consistency (Cronbach’s alpha = 0.91), to assess the AF knowledge of the general population in Australia [9]. Section two, consisting of six items, collected sociodemographic information.

The 27-item survey instrument was assessed for face validity by four content experts, who were: a cardiologist, a professor of cardiovascular nursing, an advanced practice nurse, and a researcher involved in developing content for AF patient education. The items were reviewed for relevance, appropriateness and readability [17]. Following discussion among the experts, the original AFKAT items were deemed fit for this study. Hence, no revisions were required.

### Data collection and management

All survey responses were collected on Qualtrics, which required a unique username and password to gain access. Upon completion of data collection, the survey responses were exported into a Microsoft (MS) Excel file format. All participants’ personal data required for reimbursement purposes were exported into a separate password-protected MS Excel file. All personal data information was deleted after the participants were successfully reimbursed. The MS Excel file containing the anonymous survey responses pertaining to the AFKAT and sociodemographic information were then subsequently imported to IBM SPSS [18] for statistical analyses.

### Data analysis

The IBM SPSS Statistics for Windows Version 27.0 [18] was used for all statistical analyses. Descriptive analyses were used to present the participants’ sociodemographic characteristics (frequency and percentage) and their AF knowledge scores across the five domains [mean percentage and standard deviation (SD)]. One-way analysis of variance (ANOVA) was used to identify differences in AF knowledge across subgroups of participants. Cronbach’s alpha was also computed to evaluate the internal consistency of the AFKAT. The level of statistical significance was set at *p*-value less than 0.05.

### Ethical considerations

All research activities pertaining to this study received approval from the National University of Singapore’s Institutional Review Board (NUS-IRB-2021-616).

## Results

### Participant characteristics

A total of 838 people attempted the survey but 620 completed it (Table 1). The participants took a mean time of 4.0 min (SD 2.4 min) to complete the online survey. Close to two-thirds of the participants were between the ages 21 to 40 years (64.5%) and female (60%). Majority were Chinese (85.3%) and lived in Singapore (98.4%). Most participants had at least a degree as their highest level of education (64.7%). The median monthly household income was between 2,500 and 6,000 SGD (~ 1,825 to 4,380USD) (41.5%) which the largest proportion of participants belonged to.

**Table 1:** Demographic characteristic of participants (n = 620)

Demographic characteristic	n	%
**Age (years)**		
21 to 30	217	35.0
31 to 40	183	29.5
41 to 50	82	13.2
51 to 60	74	11.9
61 to 70	55	8.9
71 and above	9	1.5
**Gender**		
Female	372	60.0
Male	248	40.0
**Ethnicity**		
Chinese	529	85.3
Malay	50	8.1
Indian	25	4.0
Others	16	2.6
**Highest level of education**		
No formal education	3	0.5
Primary School	2	0.3
Secondary School	64	10.3
Junior College/Pre-University	29	4.7
Diploma	121	19.5
Degree	313	50.5
Masters or Post-graduate degree	88	14.2
**Monthly household income** ^**†**^ **[Singapore dollars (SGD)** ^*^ **]**		
Less than 2,500	76	12.3
2,500 to 6,000	257	41.5
6,000 to 10,000	130	21.0
10,000 to 15,000	84	13.5
Greater than 15,000	73	11.8
**Country of residence**		
Singapore	610	98.4
Malaysia	4	0.6
Middle east	2	0.3
Canada	1	0.2
Others (not disclosed)	3	0.5

^**†**^ Median monthly household income of resident employed households in 2021 = 9,520 SGD

^*^ 1 SGD = 0.73 USD

### AF knowledge scores

The participants achieved a mean percentage score of 63.3 (SD 26.0) for their AF knowledge (Table 2). Across the five domains of the AFKAT, the participants scored best in identifying the risk factors of AF [mean percentage score 75.4 (SD 36.7)] and detection of AF [mean percentage score 75.0 (SD 32.0)] (Table 3). The participants also attained moderately high scores in the management of AF [mean percentage score 71.0 (SD 33.80)]. The participants scored worst in knowledge pertaining to basic information about AF [mean percentage score 60.7 (SD 28.2)] and prevention of AF [mean percentage score 53.0 [SD 35.7]).

**Table 2:** General public AF knowledge scores

	Mean Percentage score (SD)	F statistics	*p*-value
**Total** (n = 620)	63.3 (26.0)		
**Gender**			
Female (n = 372)	64.3 (25.2)	1.443	0.230
Male (n = 248)	61.8 (27.2)		
**Age** (years)			
21 to 30 (n = 217)	60.9 (30.2)	1.469	0.810
31 to 40 (n = 183)	62.7 (26.2)		
41 to 50 (n = 82)	64.6 (22.2)		
51 to 60 (n = 74)	65.4 (22.1)		
61 and above (n = 64)	69.2 (16.1)		
**Highest education level**			
No formal education/Primary school/Secondary school (n = 69)	61.0 (23.6)	1.741	0.157
Junior College/Pre-University/Diploma (n = 150)	61.6 (26.8)		
Degree (n = 313)	63.1 (27.3)		
Masters or Post-graduate Degree (n = 88)	68.8 (21.0)		
**Monthly household income** (Singapore dollars)			
Less than 2,500 (n = 76)	64.1 (23.2)	0.838	0.501
2,500 to 6,000 (n = 257)	63.9 (26.3)		
6,000 to 10,000 (n = 130)	61.3 (25.8)		
10,000 to 15,000 (n = 84)	60.8 (28.0)		
Greater than 15,000 (n = 73)	67.1 (26.0)		
**Country of Residence**			
Singapore (n = 610)	63.2 (26.1)	0.413	0.521
Others (n = 10)	68.6 (12.9)		

One-way ANOVA was done to examine the associations between the participants’ characteristics and their knowledge of AF (Table 2). There were no statistically significant differences in the AF knowledge scores across the various sociodemographic subgroups, i.e. gender, age, highest education level, monthly household income and country of residence. The Cronbach’s alpha of the AFKAT in this study was 0.952.

**Table 3:** General public AF knowledge scores across domains

AFKAT^a^ domain	Mean percentage (SD)
Basic AF information (9 items)	60.7 (28.2)
Risk factors (2 items)	75.4 (36.7)
Detection (4 items)	75.0 (32.0)
Prevention (2 items)	53.0 (35.7)
Management (4 items)	71.0 (33.8)
Total (across the 5 domains)	63.3 (26.0)

^a^ AFKAT: Atrial Fibrillation Knowledge Assessment Tool

## Discussion

To the best of our knowledge, this cross-sectional study is the first to employ digital marketing strategies on Facebook to gather responses from the public regarding their knowledge of AF. Elucidated in this study is the public’s moderately high AF knowledge scores [mean percentage score of 63.3 (SD 26.0)], which did not vary with selected demographic factors.

At present, there are several survey instruments developed to assess knowledge of AF. However, a large proportion of them were developed for patients [19]. The length of existing patient AF knowledge assessment surveys ranges from eight [20] to 58 items [21] but most of them have at least 31 items [19]. These patient AF knowledge assessment surveys may not be suitable for the general public because apart from assessing disease knowledge, they also include items examining their priorities for treatment, attitudes and behaviour towards living with AF. These items may not be relevant to one who has not been previously diagnosed with AF. Wendelboe et al. [10] developed a survey instrument to assess public awareness of AF. However, they included items to assess the participants’ awareness of other thrombotic diseases (stroke, myocardial infarction, pulmonary embolism, thrombosis, and deep vein thrombosis) and common non-thrombotic diseases (hypertension, diabetes, breast cancer, prostate cancer, and HIV/AIDS). This was done to compare the participants’ awareness of AF with that of other diseases. Considering the inclusion of these additional items and also lack of reporting of its reliability and validity, we decided not to use this instrument for this study.

We chose Abubakar et al.’s 21-item AFKAT to fulfil the aim of this study as it had relatively fewer items and assessed AF knowledge without assuming that the subject had pre-existing AF. Moreover, when administered on Australian subjects, the AFKAT had good internal consistency (Cronbach’s alpha = 0.910). When tested in our study context which comprised mostly of Singapore residents (98.4%), the AFKAT also displayed good internal consistency (Cronbach’s alpha = 0.95). In the original study, general public participants took an average of 2.5 min to complete the survey. In comparison, our study participants took a mean time of 4.0 min (SD 2.4 min) to complete the survey. The additional 1.5 min may be attributed to time spent reading the participant information sheet before attempting the survey and for leaving contact information for reimbursement purposes. Nonetheless, the time required to complete this survey was well under the recommended 10 min for online surveys [22].

When the AFKAT was administered on participants from the Australian general public, the mean percentage score was 53.4 (SD 27.7). In our study, the participants from the general public achieved a higher mean percentage score of 63.3 (SD 26.0). The sociodemographic information of the participants from the Australian general public was not disclosed in the original study [9]. As such, it was not possible to draw definitive conclusions on the difference in AF knowledge scores between the two study populations.

In this study, digital marketing strategies were employed on Facebook to recruit participants. Such web-based strategy using social media has gained interests among researchers as it is perceived to potentially reach general and specific study populations [23–25]. In particular, the utilisation of Facebook for digital marketing can effectively target a specific demographic profile and reach a diverse audience within a vast geographic location [26]. Furthermore, it has the ability to access individuals who may not actively seek out the information and therefore remain inaccessible through the use of Google AdWords [26]. This is due to AdWords’ reliance on keyword searches initiated by the user, which limits its ability to reach individuals outside of this framework [27]. Nevertheless, the study population comprised predominantly young individuals, with 56.4% of participants aged 40 years or younger. This phenomenon of recruiting larger proportions of younger individuals was also observed in earlier studies that examined the effectiveness of digital marketing on Facebook for recruiting participants to health research-related studies [25, 28, 29].

While Facebook is used across a wide range of age groups, younger adults tend to be more engaged and active on the platform than older adults [30]. This may suggest that advertising campaigns targeting younger individuals may be more effective than those targeting older individuals, and could potentially be less expensive as a result of higher engagement rates [23]. Nonetheless, it may still be worthwhile to have reached out to a younger population in our study because the risk of AF is not only associated with age. The risk of AF is also positively associated with factors such as BMI, history of myocardial infarction or stroke and high alcohol consumption [31], and these factors are modifiable. Hence, increasing awareness of AF in younger individuals provides us the opportunity to reduce their risk of developing AF later in life. Between the age of 40 to 69 years, the risk of AF is at least 37% for every 5 kg/m^2^ increase in BMI [31]. In consideration of that, individuals, including younger adults, who struggle with weight management are at risk of AF.

The moderately high AF knowledge scores obtained by our study participants may be attributed to their education level. Most (84.2%) of the participants’ high level of education was a Diploma. Compared with people with no or low levels of education, people with higher education levels have been shown to have better knowledge on general health, which enhances their awareness of health promotion behaviours [32]. Even though there was no statistically significant difference in the AF knowledge scores across the different age groups, there was a slight trend suggesting that older individuals had better AF knowledge scores than younger individuals [mean percentage score 60.9 (age group 21 to 30 years) versus mean percentage score 69.2 (age group 61 and above)]. Young adults in Singapore are less likely than older adults to take part in screening of chronic diseases [33, 34], hence, they may not be cognizant of heart rhythm disorders such AF. In contrast, older adults, especially aged 61 years and above, have a greater likelihood of living with at least one chronic disease and have regular medical reviews with their primary care provider [35]. During which, their primary care providers may have had the opportunity to provide education on AF.

However, as important as improving public knowledge of AF is, it is not the end goal. The focus should be on improving the uptake of preventive treatments as a measure to reduce stroke occurrence. Therefore, better AF detection rates and timely initiation of stroke-preventive treatments are essential metrics. Abubakar et al. (2019) suggested a framework based on the Capability, Opportunity, and Motivation Behaviour (COM-B) model to guide the design of interventions to improve the early detection of AF in the community [15]. The framework outlines the importance of improving people’s psychological and physical capabilities to engage in AF screening behaviour. Factors involved in improving these psychological and physical capabilities include AF knowledge, education level, cognitive function, and ability to respond to AF symptoms appropriately. Most of which is directly impacted by assessment of knowledge and health awareness campaigns [15].

Nevertheless, healthcare providers’ use of AF screening and diagnosis accompanied by accurate dispensation of treatment, advice and referral is just as important [36]. Health institutions play pivotal roles in supporting public education outreach and clinical decision support. Apart from implementing community-based AF screening for members of the general public, streamlining the community-based outreach and screening programmes to existing healthcare facilities can encourage sustainability [15]. This also ensures that people who have been newly diagnosed with AF undergo further diagnostic investigations and receive prompt treatment.

In our study, even though the participants achieved moderately good AF knowledge scores, they seemed to be less informed about measures to prevent AF (mean percentage score 53.0%). To curtail AF-related stroke burden, besides improving awareness of AF, preventive and therapeutic strategies must also be employed [37]. For instance, at the patient level, attempts to delay the development of AF by managing risk factors, encouraging healthy weight loss, increasing physical activity, and treating comorbidities should be advocated for. The term “atrial fibrillation” or “AFib” should be as easily recognised by the public as “cancer”, “heart attack” and “stroke”.

At policy and institutional levels, issues pertaining to access to health screening and subsequent care, prescribing of medications, adherence and persistence of treatment have to be addressed as well. It was shown that physicians may know that AF patients need be anticoagulated but this knowledge is not translated into practice [38]. Multifaceted, collective efforts among policymakers, health institutions, governmental organisations, professional societies, healthcare providers and patients are required to augment the use of stroke-preventive interventions among patients with AF [37].

### Practical implications

The utility of social media in reaching the general public was illustrated through this study. Apart from the conventional, in-person, public education outreach, social media marketing could be used to target the population at risk of AF. Nonetheless, as alluded to earlier, public education outreach and community-based AF screening should be helmed by health institutions to ensure individuals at risk of AF gets the necessary referrals for consults and investigations in a timely manner. With regards to developing content or intervention for AF public education outreach and campaigns, it may be propitious to gather patient and public involvement. These groups of people may provide valuable insights to ensuring that the content or intervention is easily understood. Lastly, further inquiry can be made to compare the impact and cost-effectiveness of running AF education campaigns using digital marketing with conventional, in-person, public outreach.

### Limitations

Some limitations of this study deserve comment. First, the survey was distributed online via Facebook, restricting it to respondents with internet access and Facebook accounts. Such individuals tend to have higher education levels and also younger in age. In addition, the online survey was posted on NUHCS Facebook page. People who visit the site would probably have health-seeking behaviours or interest in cardiovascular health issues. Digital marketing strategies were employed to “push” the survey post to members of the public and not just followers and visitors of the NUHCS Facebook page. Nonetheless, intermittent checks on demographic profile of our study participants should have been done during the data collection period so that modifications could be made to the Facebook’s advertising targeting algorithm to reduce the “push” to younger individuals.

## Conclusions

Members of the public recruited from Facebook and via digital marketing had moderately good knowledge of AF. However, public awareness pertaining to preventing AF has potential for improvement. The utility of social media in reaching the general public was illustrated through this study.

## Electronic supplementary material

Below is the link to the electronic supplementary material.

Supplementary Material 1

## Data Availability

The data presented in this study are available on request from the corresponding author. The data are not publicly available due to the university’s data sharing policy.

## Abbreviations

AF: Atrial Fibrillation
AFKAT: Atrial Fibrillation Knowledge Assessment Tool
ANOVA: Analysis of Variance
COM-B: Capability, Opportunity, and Motivation Behaviour
ECG: Electrocardiogram
NUHCS: National University Heart Centre, Singapore
URL: Uniform Resource Locator
