# Supplementary material for: Assessment of the general public’s knowledge of atrial fibrillation through social media: a cross-sectional study
Source: BMC Nurs. 2023 Jun 16;22:207. doi: 10.1186/s12912-023-01378-7 (PMC10273733; doi:10.1186/s12912-023-01378-7)
Supplement: Supplementary file 1 — Supplementary Material 1 [file 12912_2023_1378_MOESM1_ESM.docx]

| Supplementary File 1: General Public’s Atrial Fibrillation Knowledge Assessment Tool  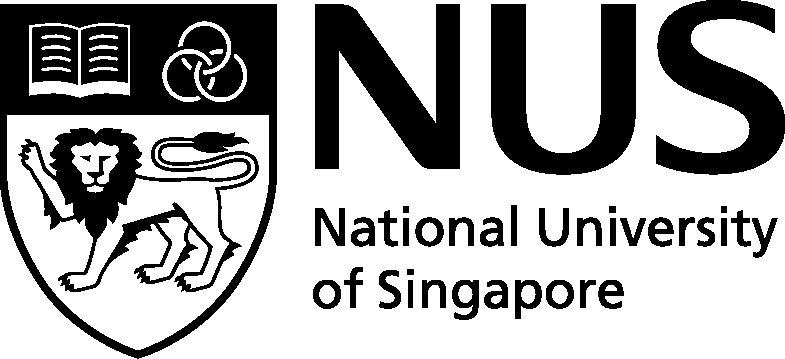 | |  |  |
| --- | --- | --- | --- |
|  | | **Option** | **Answer** |
| 1 | Atrial fibrillation is a medical condition where the heart beats slower than normal | True / False / Don’t know | False |
| 2 | Atrial fibrillation may cause blood clots in the heart. | True / False / Don’t know | True |
| 3 | Episodes of atrial fibrillation are predictable. | True / False / Don’t know | False |
| 4 | People with atrial fibrillation can still have an active life. | True / False / Don’t know | True |
| 5 | Atrial fibrillation can only be treated with surgery. | True / False / Don’t know | False |
| 6 | Episodes of atrial fibrillation can be recurrent. | True / False / Don’t know | True |
| 7 | Early diagnosis and management of atrial fibrillation can prevent stroke. | True / False / Don’t know | True |
| 8 | Low blood pressure increases the risk of developing atrial fibrillation. | True / False / Don’t know | False |
| 9 | Atrial fibrillation significantly increases the risk of stroke. | True / False / Don’t know | True |
| 10 | Atrial fibrillation occurs only in people with prior signs of heart disease. | True / False / Don’t know | False |
| 11 | Shortness of breath and fainting can be potential symptoms of atrial fibrillation. | True / False / Don’t know | True |
| 12 | Atrial fibrillation occurs only in old age. | True / False / Don’t know | False |
| 13 | Someone could have atrial fibrillation without having any symptoms. | True / False / Don’t know | True |
| 14 | Symptoms of atrial fibrillation may be occasional, persistent, or permanent. | True / False / Don’t know | True |
| 15 | Atrial fibrillation usually has major psychological effects on people’s lives. | True / False / Don’t know | False |
| 16 | The risk of developing atrial fibrillation can be reduced with lifestyle changes. | True / False / Don’t know | True |
| 17 | Atrial fibrillation can be detected by checking the regularity of the pulse. | True / False / Don’t know | True |
| 18 | Screening for atrial fibrillation is safe. | True / False / Don’t know | True |
| 19 | Once present, atrial fibrillation is always a lifelong condition. | True / False / Don’t know | False |
| 20 | Atrial fibrillation can be treated with medications. | True / False / Don’t know | True |
| 21 | Anticoagulants (“blood thinners”) are often used to reduce the risk of stroke in people with atrial fibrillation. | True / False / Don’t know | True |

**Demographic information**

22. Gender:

1. Female
2. Male

23. Age (years):

1. 21 to 30
2. 31 to 40
3. 41 to 50
4. 51 to 60
5. 60 to 70
6. 71 & above

24. Ethnicity:

1. Chinese
2. Malay
3. Indian
4. Others (please specify):

25. Highest education level:

1. No formal education
2. Primary school
3. Secondary school
4. Junior College/Pre-University
5. Diploma
6. Degree
7. Masters or post-graduate degree

26. Household monthly income:

1. Less than SGD 2500
2. SGD 2500 to SGD 6000
3. SGD 6000 to SGD 10,000
4. SGD 10,000 to SGD 15,000
5. More than SGD 15,000

27. Country of residence:

1. Singapore
2. Others (Please specify):

**Do you consent to be contacted via your mobile no. for reimbursement purposes?** Your name and contact details will be deleted immediately after reimbursement have been completed.

Yes or No (Please select)

**If yes,**

**Name:**

**Mobile number:**
